# Thrombotic Microangiopathy Presenting With Multisystem Involvement and Acute Neurologic Deficits in a 61-Year-Old Female: A Case of Acquired Thrombotic Thrombocytopenic Purpura

**DOI:** 10.7759/cureus.114232

**Published:** 2026-08-09

**Authors:** Timmer Verhaegh, Anais Panossian, Fady Michael, Tanvir Rahman, Aram A Namavar, Zubair Ilyas

**Affiliations:** 1 Internal Medicine, University of California, Los Angeles (UCLA), Los Angeles, USA; 2 Internal Medicine, Western University of Health Sciences, Pomona, USA; 3 Medicine, University of California, Los Angeles (UCLA), Los Angeles, USA; 4 Medicine, University of California, Los Angeles (UCLA) Health, Los Angeles, USA

**Keywords:** adamts13 deficiency, multisystem involvement, plasma exchange, thrombotic microangiopathy, thrombotic thrombocytopenic purpura

## Abstract

Thrombotic microangiopathy (TMA) is a rare, life-threatening hematologic syndrome characterized by microangiopathic hemolytic anemia, thrombocytopenia, and end-organ dysfunction. Among TMAs, thrombotic thrombocytopenic purpura (TTP), an emergent subtype caused by severe deficiency of a disintegrin and metalloproteinase with thrombospondin type 1 motif, member 13 (ADAMTS13), requires urgent recognition and prompt treatment. We present a 61-year-old woman who initially presented with abdominal pain and hematuria and was treated for a presumed urinary tract infection. She later developed chest pain, unilateral weakness, melena, and petechiae. Laboratory evaluation revealed severe anemia, thrombocytopenia, acute kidney injury, elevated lactate dehydrogenase, and schistocytes on peripheral smear, consistent with microangiopathic hemolysis. Urgent hematologic evaluation raised concern for TTP versus hemolytic uremic syndrome. Her PLASMIC score was high risk, prompting empiric initiation of plasma exchange and corticosteroids prior to confirmatory testing. Brain magnetic resonance imaging (MRI) demonstrated punctate acute infarcts in the right parietal lobe. ADAMTS13 activity returned <10%, confirming acquired TTP. The patient improved with plasma exchange, corticosteroids, and rituximab, and was discharged in stable condition with outpatient follow-up. This case highlights the importance of early recognition, use of clinical prediction tools, and prompt treatment of TTP in patients with multisystem involvement and neurologic deficits to reduce morbidity and mortality.

## Introduction

Thrombotic microangiopathy (TMA) encompasses a group of disorders characterized by microangiopathic hemolytic anemia, thrombocytopenia, and variable degrees of organ dysfunction [1]. Thrombotic thrombocytopenic purpura (TTP) is a rare but critical form of TMA caused by severe deficiency of a disintegrin and metalloproteinase with thrombospondin type 1 motif, member 13 (ADAMTS13) - a von Willebrand factor-cleaving protease, resulting in widespread microvascular platelet-rich thrombi and tissue ischemia [2].

Untreated TTP carries a mortality rate exceeding 90%; however, early initiation of plasma exchange has dramatically improved survival [3]. Diagnosis is often challenging due to heterogeneous presentations involving neurologic, renal, gastrointestinal, and cardiac systems. Patients often present with nonspecific symptoms, such as fatigue, weakness, abdominal pain, confusion, or transient neurologic deficits before the classic pentad develops, making early recognition particularly challenging. Clinical prediction tools, such as the PLASMIC score, are valuable in estimating the likelihood of severe ADAMTS13 deficiency and guiding early empiric therapy when laboratory confirmation is delayed [4,5].

We present a case of acquired TTP with multisystem involvement and acute neurologic deficits in which early recognition and multidisciplinary care facilitated a favorable clinical outcome.

## Case presentation

A 61-year-old female with a history of hypertension, prediabetes, and hepatic steatosis initially presented with abdominal pain, nausea, and hematuria. She was discharged on oral ciprofloxacin for presumed urinary tract infection. One week later, she returned with chest pain, unilateral weakness, petechiae, melena, and persistent hematuria. She also reported recent aspirin use due to prior concern for cerebrovascular disease.

On presentation, she was hemodynamically stable. Physical examination revealed diffuse petechiae and focal unilateral weakness. Laboratory studies demonstrated decreased hemoglobin of 7.9 g/dL, decreased platelet count of 25 × 10⁹/L, borderline creatinine of 2.1 mg/dL, elevated lactate dehydrogenase of 931 U/L, indirect hyperbilirubinemia of 1.2 mg/dL, and low haptoglobin of <10 mg/dL. Peripheral smear revealed schistocytes, consistent with microangiopathic hemolytic anemia.

Computed tomography of the chest, abdomen, and brain revealed no acute abnormalities. Aspirin and ciprofloxacin were discontinued.

Because the patient's initial presentation consisted of generalized weakness, dysarthria, urinary symptoms, and altered mental status, the initial differential diagnosis included urinary tract infection, acute ischemic stroke, sepsis, and other causes of TMA. The constellation of progressive thrombocytopenia, hemolytic anemia, and neurologic findings subsequently shifted clinical suspicion toward TTP.

Given concern for TMA, hematology was consulted, and the patient was transferred to the intensive care unit. Her laboratory findings are summarized in Table 1. Her PLASMIC score was subsequently calculated as 6 (Table 2) (platelet count <30 × 10⁹/L, hemolysis, no active cancer, no history of transplant, mean corpuscular volume (MCV) <90 fL, international normalized ratio (INR) <1.5), consistent with high risk for severe ADAMTS13 deficiency. The PLASMIC score was calculated using the published criteria originally described by Bendapudi et al. and is publicly available through MDCalc [4].

**Table 1:** Initial laboratory findings The laboratory findings that are most supportive of thrombotic thrombocytopenic purpura include severe thrombocytopenia, schistocytes on peripheral blood smear, elevated lactate dehydrogenase, and decreased haptoglobin.

Parameter	Result	Reference Range
Hemoglobin	7.9 g/dL	12.0–16.0 g/dL
Platelet Count	25 × 10⁹/L	150–400 × 10⁹/L
Creatinine	2.1 mg/dL	0.6–1.3 mg/dL
Lactate Dehydrogenase (LDH)	931 U/L	140–280 U/L
Indirect Bilirubin	1.2 mg/dL	0.2–0.8 mg/dL
Haptoglobin	<10 mg/dL	30–200 mg/dL
Peripheral Smear	Schistocytes present	None

**Table 2:** PLASMIC score assessment

Criterion	Finding	Points
Platelet count <30 × 10⁹/L	Yes	1
Hemolysis (↑ LDH, ↓ haptoglobin, schistocytes)	Yes	1
No active cancer	Yes	1
No history of solid organ or stem cell transplant	Yes	1
Mean corpuscular volume (MCV) <90 fL	Yes (85.2 fL)	1
International normalized ratio (INR) <1.5	Yes (1.1)	1
Creatinine <2.0 mg/dL	No (borderline)	0
Total PLASMIC Score: 6 (High Risk for Severe ADAMTS13 Deficiency)		

Given the patient's high-risk PLASMIC score (6 points) and strong clinical suspicion for acquired TTP, empiric treatment was initiated immediately without waiting for confirmatory ADAMTS13 testing. Empiric treatment with daily plasma exchange and high-dose intravenous methylprednisolone was initiated on hospital day one. ADAMTS13 activity, complement testing, and additional diagnostic studies were sent; however, therapy was not delayed because of the high mortality associated with untreated TTP.

**Figure 1:**
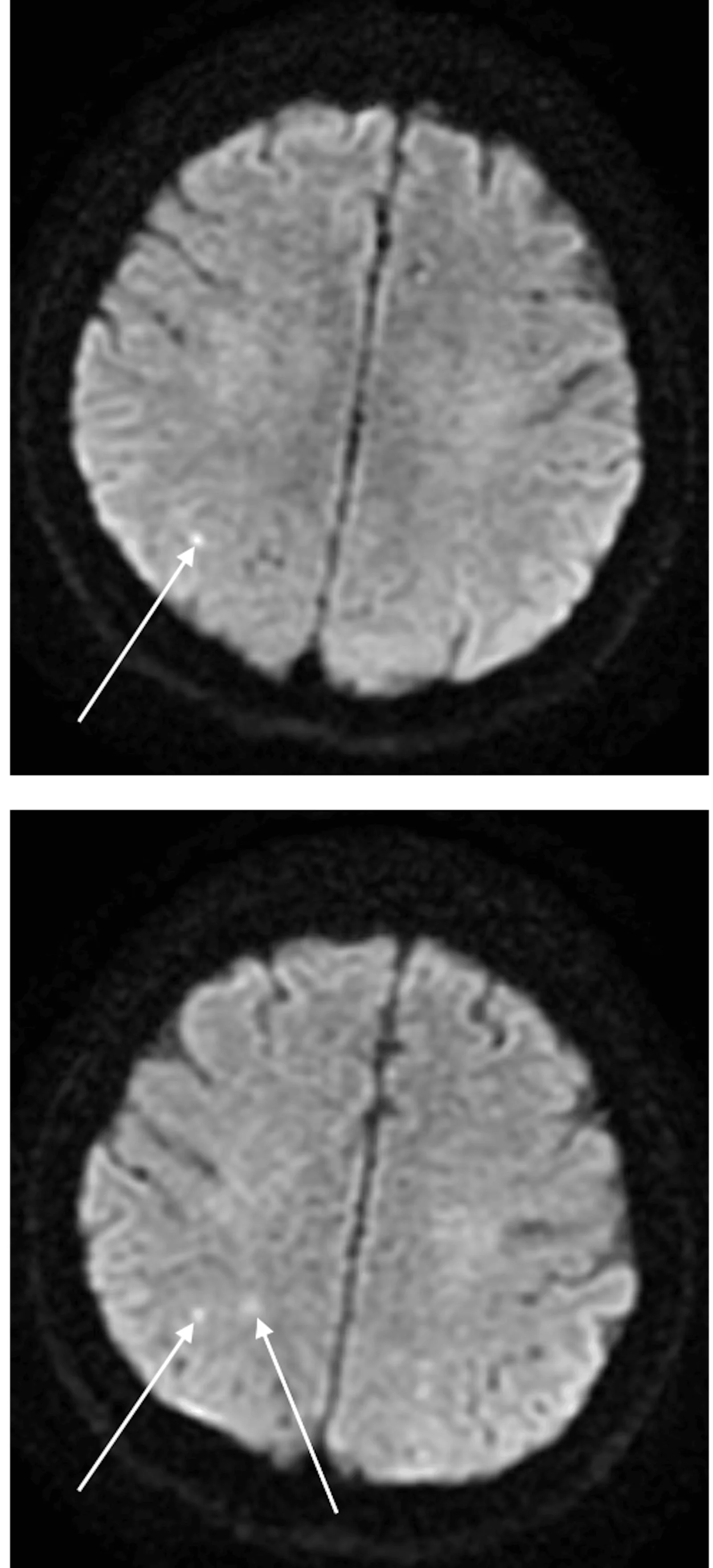
Brain magnetic resonance imaging demonstrating punctate acute infarcts in the right parietal lobe (arrows), consistent with microvascular ischemia in thrombotic thrombocytopenic purpura

Neurology evaluation prompted brain magnetic resonance imaging (MRI), which demonstrated multiple punctate areas of restricted diffusion in the right parietal lobe on diffusion-weighted imaging (DWI), with corresponding apparent diffusion coefficient (ADC) changes, consistent with acute microvascular ischemic infarcts (Figure 1).

Nephrology attributed the acute kidney injury to acute tubular necrosis, as renal function improved with supportive care. Gastroenterology determined that melena was secondary to thrombocytopenia-related mucosal bleeding.

ADAMTS13 activity subsequently returned <10%, confirming acquired TTP. Eculizumab was considered but not initiated. Over the hospital course, the patient's platelet count progressively improved, accompanied by normalization of platelet, lactate dehydrogenase, and haptoglobin levels following plasma exchange and immunosuppressive therapy (Table 3). Plasma exchange was discontinued after clinical stabilization. She was transitioned to an oral prednisone taper and initiated on weekly rituximab infusions.

**Table 3:** Hematologic response to therapy during hospitalization Serial laboratory values demonstrating hematologic recovery following initiation on hospital day one of plasma exchange and immunosuppressive therapy for acquired thrombotic thrombocytopenic purpura.

Hospital Day	Platelets	LDH	Haptoglobin
Day 1	25 × 10⁹/L	931 U/L	<10 mg/dL
Day 2	31 × 10⁹/L	N/A	N/A
Day 3	55 × 10⁹/L	298 U/L	78 mg/dL
Day 4	83 × 10⁹/L	N/A	N/A
Day 5	132 × 10⁹/L	227 U/L	89 mg/dL
Day 6	203 × 10⁹/L	200 U/L	155 mg/dL
Day 7	242 × 10⁹/L	177 U/L	192 mg/dL

Aspirin was resumed after platelet recovery for secondary stroke prevention. The patient was discharged in stable condition with a plan for close outpatient follow-up. Long-term neurologic recovery and relapse outcomes are unavailable because follow-up occurred outside the institution.

## Discussion

TTP is a hematologic emergency requiring immediate recognition and treatment. It is characterized by microangiopathic hemolytic anemia, severe thrombocytopenia, and organ dysfunction due to widespread platelet-rich microthrombi [3,6].

This case illustrates the heterogeneous presentation of TTP, beginning with nonspecific symptoms and progressing to multisystem involvement, including neurologic deficits, renal dysfunction, and gastrointestinal bleeding. Such variability can delay diagnosis and highlights the importance of maintaining a high index of suspicion.

The PLASMIC score is a validated clinical tool that predicts severe ADAMTS13 deficiency and helps guide early management decisions [4,5]. In this case, a high-risk score supported the prompt initiation of plasma exchange prior to confirmatory testing, which is critical in reducing mortality.

Several findings represented key diagnostic turning points in this case. The presence of schistocytes on a peripheral blood smear, together with severe thrombocytopenia, elevated lactate dehydrogenase, undetectable haptoglobin, and a high-risk PLASMIC score, rapidly increased suspicion for TTP despite the patient's initially nonspecific presentation. This prompted immediate plasma exchange before confirmatory ADAMTS13 testing became available, illustrating the importance of recognizing evolving TMA early in the disease course.

Although renal involvement is more commonly associated with hemolytic uremic syndrome, mild to moderate acute kidney injury can occur in TTP, complicating differentiation between TMA subtypes [1]. Severe ADAMTS13 deficiency (<10%) remains the most specific diagnostic marker for TTP [2].

Neurologic involvement occurs in approximately one-half to two-thirds of patients with acquired TTP and may manifest as headache, confusion, focal neurologic deficits, seizures, or ischemic stroke [6,7]. These findings reflect the underlying microvascular thrombosis affecting cerebral circulation.

Standard treatment includes plasma exchange and immunosuppression with corticosteroids, with rituximab increasingly used to reduce the risk of relapse [3,7,8]. More recently, caplacizumab, a monoclonal antibody targeting von Willebrand factor, has emerged as an effective adjunctive therapy that reduces time to platelet recovery and thrombotic complications [9,10].

In the present case, plasma exchange was initiated before ADAMTS13 confirmation because delaying therapy in suspected TTP is associated with substantially increased mortality. Multidisciplinary collaboration among hematology, neurology, nephrology, and gastroenterology facilitated rapid diagnosis, exclusion of competing etiologies, and timely initiation of life-saving therapy. Although long-term neurologic recovery and relapse outcomes were unavailable, the patient demonstrated clinical stabilization with hematologic recovery prior to discharge.

## Conclusions

This case underscores the importance of early recognition, empiric treatment, and coordinated multidisciplinary care in improving outcomes in TTP. TTP should be suspected in patients presenting with thrombocytopenia, hemolytic anemia, and multisystem involvement, particularly when neurologic symptoms are present, even when the initial presentation is nonspecific. Clinical tools, such as the PLASMIC score, can aid in early risk stratification and support prompt initiation of life-saving therapy while awaiting confirmatory ADAMTS13 testing. Continued multidisciplinary management and close follow-up remain essential because of the risk of relapse and persistent neurologic sequelae.
